# The Illinois State Board of Health and Reform in Medical Education

**Published:** 1885-06

**Authors:** 


### Editorial.

### The Illinois State Board of Health and Reform in Medical Education.

The State of Illinois is to be congratulated upon the possession of a Model Board of Health; certainly this must be admitted if we adopt, as the standard of judgment, "by their fruits ye shall know them," in estimating the work accomplished by the several State Health Boards. The opportunities of the Illinois Board, it is true, have been somewhat greater than the others, for the reason that its powers were of a two-fold nature. In addition to the ordinary control of public sanitation, as exercised by boards of health in general, it also performs the functions of State Medical Examining Board, thus having control, to a certain extent, of medical educational interests within the State.

The capable and satisfactory administration of the laws relating to public health, and the practice of medicine in Illinois, have been the subject of much favorable comment, both in medical and lay circles, for some time past; and we desire to add our testimony in appreciation of the good work which is being accomplished, in both channels named, by the excellent Board of Health in that State. But it is our present purpose to speak more particularly of the educational work which is being wrought under its auspices. It is in this direction that its acts and offices are felt outside the State of Illinois; for there is not a medical school in the land which does not feel the influence, in some degree, of the attempt which the board is making to improve the educational standard in medicine. To show how far-reaching this effort on the part of a single State is, the following circular, which has been addressed to the Secretary of all medical colleges in the United States, is here introduced:

ILLINOIS STATE BOARD OF HEALTH,
OFFICE OF THE SECRETARY, SPRINGFIELD, May 4, 1885.

DOCTOR:

At the regular quarterly meeting of the ILLINOIS STATE BOARD OF HEALTH, held in the city of Chicago, April 16-17, 1885, the following preamble and resolutions were unanimously adopted:

CONCERNING MEDICAL EDUCATION.

WHEREAS, Many medical colleges do publicly announce that an entrance examination of candidates for admission to their lecture courses will be exacted, and do honestly and impartially enforce such examination; while, on the other hand, a number of schools either avoid making such announcement, or evade the practical enforcement of any requirement of general education preliminary to the study of medicine; and

WHEREAS, These conflicting practices result in lowering the standard of medical education by attracting to a certain class of schools, students who are poorly prepared for the study of medicine: Therefore, be it

*Resolved*, That, in order to secure the recognition of its diplomas as in good standing for the purposes of the Medical-Practice Act in this State, it is necessary that each college shall distinctly state in its Annual Announcement that the conditions of admission to its classes are: 1. Credible certificates of good moral character. 2. Diploma of graduation from a good literary and scientific college or high school, or a first-grade teacher's certificate. Or, lacking this, a thorough examination in the branches of a good English education, including mathematics, English composition, and elementary physics or natural philosophy.

*Resolved*, That the Secretary of the Board be, and hereby is, instructed to furnish a copy of the foregoing preamble and resolutions to the Dean or Secretary of every medical college, and to the editors of medical journals, in the United States.

*Resolved*, That since the publication of the names and addresses of matriculates is desirable for purposes of information, the Secretary be authorized to request of all colleges desirous of being accounted in good standing in this State, that they publish in their successive annual announcements complete lists of the matriculates, as well as of the graduates, of each immediately preceding session.

Very respectfully,

JOHN H. RAUCH, M. D., *Secretary.*

A careful perusal of this circular will give the medical student, who intends to locate after graduation in Illinois, but who prefers to pursue his studies in some college outside of that State, just the information he requires in order to become legally qualified under its Medical-Practice Act. He will at once ob erve that it will be futile for him to obtain the degree of any college which does not conform to the above requirements. The advantages to the student of a uniform standard for entrance into, and graduation from, our medical colleges is too apparent to admit of extended argument in favor thereof; and, though it may be too much to expect the latter, it is quite within reason to hope for the former. Let the colleges agree, once for all, to demand of each matriculate a certain minimum standard entrance examination, and a great step will be taken towards the much needed reform in our system of medical education. The older colleges can afford to make this concession to the good of the cause, which all true lovers of our profession ought to have at heart; while the younger ones will, as a matter of necessity, fall into line.

It is a matter of pride that one of the medical colleges in our own city,—The Medical Department of Niagara University,—has taken such an advanced position in this matter. Organized in the interests of a higher standard for medical education, it is steadily advancing in the direction named, with a constantly growing confidence in the purity of its purposes, indicated by increased patronage and a manifest growth in public favor. The stand it has so boldly taken and successfully maintained places it in the front rank of American Medical Colleges, and entitles it to a continuance of public confidence and favor.

The attitude of the JOURNAL on the question of a State Medical Examining Board is well known. From the first, it has been an uncompromising champion of the principle of one licensing power for the whole State. It is to be deeply regretted that thus far all efforts in this direction have met defeat. We seem to have been handicapped by the medical sects—homeopathic and eclectic—which the laws of the State recognize. They demand separate examining boards, which is, of course, so impracticable a scheme that the whole plan falls temporarily to the ground. This cannot prevent the ultimate passage of a law which shall divorce the teaching and licensing power; but it provokes a delay, which is as unfortunate as it is puerile. The tend of public sentiment is, however, so decidedly in favor of such a law, that it will not brook idle or trifling dalliance with a question involving the best interests of the commonwealth; and woe be to the man, the sect, ring, or clique, that shall thwart a mature and well-grounded demand of the people on a question effecting their health, longevity and good name. If the friends of education will only stand together in this matter for a little while longer, their efforts will surely meet with that success which they so richly deserve. The great State of New York, with its vast wealth, intelligence, and almost unbounded resources, cannot afford to fall to the rear of the column in the grand march of the commonwealth towards a higher civilization—a march in which Illinois, in so far as relates to a higher medical education, now so proudly leads the van.

